# Reminiscence therapy-based care program serves as an optional nursing modality in alleviating anxiety and depression, improving quality of life in surgical prostate cancer patients

**DOI:** 10.1007/s11255-022-03282-6

**Published:** 2022-07-16

**Authors:** Ting Huang, Hongmei Su, Shi Zhang, Yawen Huang

**Affiliations:** 1 Operating Room, Renmin Hospital of Wuhan University, Central Area, Wuhan, 430060 China; 2 Operating Room, Renmin Hospital of Wuhan University, Guanggu Area, No. 99, Zhangzhidong Road, Wuhan, 430060 China; 3 Department of Ophthalmology, Wuhan No. 1 Hospital, No. 215, Zhongshan Avenue,, Wuhan, 430022 China

**Keywords:** Reminiscence therapy-based care program, Prostate cancer patients, Anxiety, Depression, Quality of life

## Abstract

**Purpose:**

Reminiscence therapy is reported to attenuate the psychological disorders in cancer patients, such as colorectal and lung cancer patients. However, relevant report on surgical prostate cancer patients is scarce. This study put forward a reminiscence therapy-based care program (RTCP + UC) combing reminiscence therapy with usual care (UC), and aimed to evaluate the impact of RTCP + UC on anxiety, depression, quality of life and survival in surgical prostate cancer patients.

**Methods:**

Totally, 108 prostate cancer patients receiving surgical resection were enrolled, who were subsequently randomized and allocated to the RTCP + UC group (*N* = 55) and UC group (*N* = 53) at a 1:1 ratio. Hospital Anxiety and Depression Scale (HADS) and QLQ-C30 were assessed at month M0, M3, M6, M9 and M12 during the intervention period. After intervention, patients were followed up for another 24 months to calculate disease-free survival (DFS) and overall survival (OS).

**Results:**

RTCP + UC decreased HADS-anxiety score at M9 and M12, declined HADS-depression score at M6, M9 and M12, reduced depression rate and the severity level of depression at M12, while did not affect these issues at other time points. Meanwhile, RTCP + UC enhanced the QLQ-C30 global health status score at M3, M6, M9 and M12, but did not influence the QLQ-C30 function score and QLQ-C30 symptom score at any time points. Meanwhile, RTCP + UC had no effect on the accumulating DFS and OS of surgical prostate cancer patients.

**Conclusion:**

RTCP + UC serves as an optional nursing modality in alleviating anxiety and depression, improving quality of life in surgical prostate cancer patients.

**Supplementary Information:**

The online version contains supplementary material available at 10.1007/s11255-022-03282-6.

## Introduction

Prostate cancer, characterized by dysuria, leakage of urine and erectile dysfunction, is the second-most common cancer in men over the world with estimated 1,414,259 new cases and 375,304 new deaths according to the data issued by *GLOBOCAN* in 2020 [1–3]. Even though the survival of prostate cancer patients is satisfactory with 5-year survival ranging from 42.0% to nearly 100% after surgical resection [4–6], the mental burden of surgical prostate cancer patients is still huge, which manifests as a frequent occurrence of phycological disorders (including anxiety and depression) and decreased life quality resulted from the disease itself or the treatment modalities, such as orchiectomy and androgen deprivation (ADT) [7, 8]. Meanwhile, the emergence of anxiety and depression might further cause a decrement of the survival of surgical prostate cancer patients [9]. Despite great efforts that have been made to relieve anxiety and depression, improve the quality of life, and further prolong the survival of surgical prostate cancer patients, how to figure out these problems in a preferable manner still challenges modern health care providers.

Reminiscence therapy was initially proposed for the treatment of cognition impairment in Alzheimer’s disease in the 1990s, which is mainly applied by leading the patients to recall their memories and share their experiences, etc. under the help of trained nurses [10]. Apart from the influences on cognitive impairment, over the past two decades, reminiscence therapy has also been elucidated to attenuate anxiety and depression, and improve the life quality in various cerebral diseases, including acute ischemic stroke and dementia [11, 12]. However, few cancer patients receive reminiscence therapy, and the rare studies reported on reminiscence therapy were primarily related to colorectal and lung cancers, which show that reminiscence therapy-based care program (RTCP + UC) might relieve anxiety, depression and improve the quality of life in colorectal cancer patients and lung cancer patients [13, 14], while no report suggests the same finding in the surgical prostate cancer. Thus, we conducted this randomized, controlled study to evaluate the influence of RTCP + UC on anxiety, depression, quality of life and the survival of surgical prostate cancer patients who received surgical resection.

## Methods

### Patients

After being approved by Institutional Review Board, a total of 108 prostate cancer patients who underwent surgical resection in our hospital between February 2016 and April 2018 were enrolled in this randomized and controlled study. The inclusion criteria were: (1) diagnosed as primary prostate cancer; (2) aged more than 18 years; (3) received surgical resection; (4) able to understand mandarin and communicate with others normally; (5) possessed an ability to complete study evaluation independently; (6) with normal cognitive function and understanding. The exclusion criteria were: (1) confirmed as relapsed, secondary or advanced disease at first admission to our hospital; (2) complicated with other malignant diseases; (3) with known severe psychological diseases requiring long-term pharmacological intervention; (4) had cognitive disorders or communication barriers; (5) accompanied with poorly controlled complications that seriously affected patients’ life quality and mental health; (6) treated by other modalities such as external beam radiotherapy (EBRT). After fully understanding the content of this study, all patients voluntarily signed the informed consents.

### Random allocation

Eligible patients were randomly allocated to RTCP + UC group (*N* = 55) or usual care (UC) group (*N* = 53). Block randomization method was used for assignment of patients. The allocation ratio was 1:1, and the block size was set as 4. The random assignment information of each patient was sealed in an opaque envelope which was corresponded one-to-one with the patient enrollment number. When a patient’s eligibility was confirmed, an opaque envelope with an enrollment number was assigned to the patient, who was then allocated to the corresponding group.

### Study intervention

In the UC group, patients were given usual care during the postoperative hospital stay, including routine nursing, subsequent therapy, nutrition support, prevention of infection and complications, and rehabilitation guidance, all of which were not interfered with by this study. After discharge, in addition to regular reexamination, no other care was conducted for them.

In the RTCP + UC group, routine postoperative care was given to all patients during the postoperative hospital stay, which was the same as those in the UC group. In addition to regular reexamination, the RTCP + UC was initiated from the first month after discharge. The RTCP + UC was composed of two parts: health education and reminiscence therapy (RT). Patients were invited to the rehabilitation center to receive the RTCP + UC from the first month after discharge. The RTCP + UC was performed every 2 weeks for 12 consecutive months, and carried out in the form of conversation group (each group contains 6 patients). Each RTCP + UC took 100 min, with 20 min for health education and 80 min for RT. The health education covered basic knowledge of prostate cancer, patient self-monitoring, matters needed attention, management of drugs and complications, nutrition supporting, exercise at home, and psychological health. The RT was carried out based on the following 12 topics: (1) self-introductions of personalities and their family; (2) funny things in childhood; (3) school life stories; (4) memory of hometown; (5) festival custom in your hometown; (6) romantic experiences and marriage life; (7) working experiences; (8) unforgettable travel experience; (9) an epoch-making event in one’s life; (10) favorite movie or songs; (11) talent show; (12) summary and farewell. Each conversation was led by two trained nurses in a harmonious atmosphere, and the trained nurses were responsible for motivating activities. In addition, patients were encouraged to bring their families to join in the conversation.

### Outcome evaluation within 12-month intervention period

The outcome evaluation within 12-month intervention period included anxiety status, depression status and quality of life of all patients. The time points of outcome evaluation were set as follows: baseline (M0), month 3 (M3), month 6 (M6), month 9 (M9) and month 12 (M12) after initiation of the study intervention. The anxiety status and depression status were identified using the Hospital Anxiety and Depression Scale (HADS) (Chinese version) whose psychometric properties had been validated before [15], and this questionnaire contained 2 subscales: HADS for anxiety (HADS-Anxiety) and HADS for depression (HADS-Depression). The HADS-A score or HADS-D score more than 7 was considered as anxiety or depression [16], respectively; furthermore, the severity was classified as no anxiety/depression (score 0–7), mild anxiety/depression (score 8–10), moderate anxiety/depression (score 11–14), and severe anxiety/depression (score 15–21) [17].

The quality of life was measured by the European Organization for Research and Treatment of Cancer quality of life Questionnaire-Core 30 (QLQ-C30, Version 3) (Chinese version) whose reliability and validity had been examined before [18]. The assessment of quality of life was focused on the function, symptom score and global health status in QLQ-C30. Higher QLQ-C30 function score and global health status score indicated a better physical function and overall health status, while a higher QLQ-C30 symptom score indicated a worse symptom [19].

### Survival evaluation

After 12-month intervention period, patients were routinely followed up for a total of 36 months until death. Disease status at each clinical visit or telephone connection was documented for survival evaluation. Disease-free survival (DFS) and overall survival (OS) were estimated with the use of follow-up records. In the survival analysis, patients who lost follow-up were censored on the date they were last examined or contacted.

### Statistical analysis

Assuming that the mean HADS-Anxiety score at M12 was 6.0 with standard deviation (SD) of 1.5 in RTCP + UC group and 7.0 with SD of 1.5 in UC group, respectively, the required minimum sample size should be 42 in each group at a significance (*α*) level of 0.05 and a power of 0.85. The final sample size was expanded to 54 in each group under the condition of approximately 20% dropouts. Data analysis was performed in intention-to-treat (ITT) dataset. The missing data were processed by the last observation carried forward (LOCF) method. Comparison between two groups was checked using Chi-square test, independent sample *t* test and Wilcoxon rank-sum test. Kaplan–Meier curve and log-rank test were applied for survival analysis. SPSS 26.0 statistical software (IBM, Chicago, IL, USA) and GraphPad Prism 7.01 software (GraphPad Software Inc., San Diego, CA, USA) were applied for statistical analysis and graphing. A *P* value < 0.05 was considered as statistical significance.

## Results

### Study flow

**Fig. 1:**
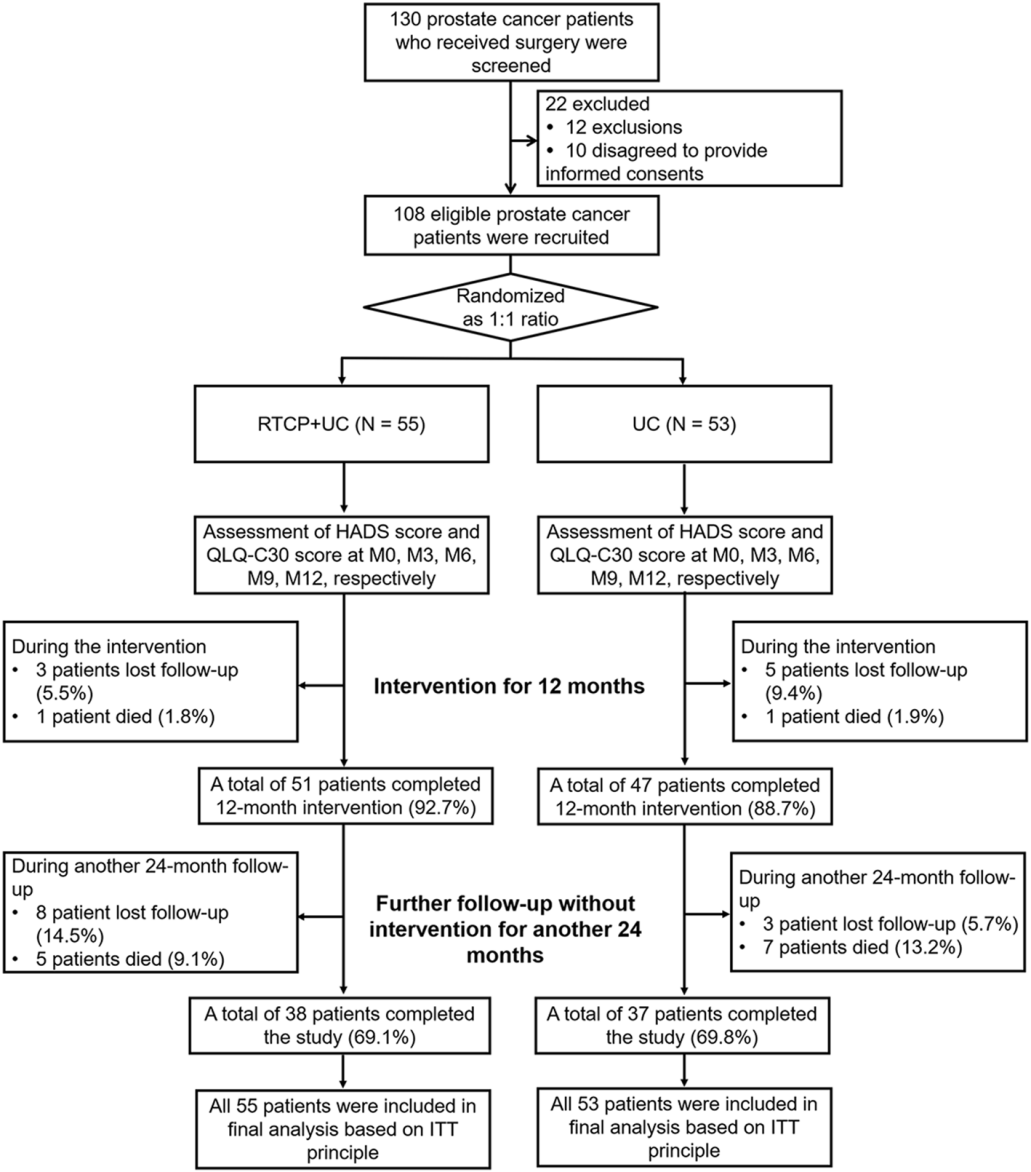
Study flow. *RTCP + UC* reminiscence therapy-based care program, *UC* usual care, *HADS-A* Hospital Anxiety and Depression Scale for anxiety, *HADS-D* Hospital Anxiety and Depression Scale for depression, *QLQ-C30* European Organization for Research and Treatment of Cancer quality of life Questionnaire-Core 30, *M* month; *ITT* intention-to-treat

A total of 130 prostate cancer patients who received surgery were screened, but 22 of them were excluded (including 12 patients who met the exclusion criteria or did not meet the inclusion criteria, and 10 patients who disagreed to provide the informed consents). The remaining 108 eligible surgical prostate cancer patients were recruited and randomly divided into the RTCP + UC group (*N* = 55) and UC group (*N* = 53) at a ratio of 1:1. Then, surgical prostate cancer patients in the RTCP + UC group and UC group received corresponding treatment for 12 months, during which 3 patients lost follow-up and 1 patient died in the RTCP + UC group, 5 patients lost follow-up and 1 patient died in the UC group. Thus, 51 patients in the RTCP + UC group and 47 patients in the UC group completed the 12-month intervention. After that, all surgical prostate cancer patients were followed up without intervention for another 24 months. During this period, 8 patients lost follow-up and 5 patients died in the RTCP + UC group. Moreover, 3 patients lost follow-up and 7 patients died in the UC group. Finally, a total of 38 patients and 37 patients completed the study in the RTCP + UC group and the UC group, respectively (Fig. 1). For the final analysis, all 55 patients and 53 patients in the RTCP + UC group and UC group were incorporated according to the ITT principle, respectively.

### Baseline characteristics

The age was 62.4 ± 9.0 years and 63.1 ± 9.4 years in the RTCP + UC and UC groups, respectively (*P* = 0.688). As to other baseline characteristics of surgical prostate cancer patients, including smoking history, hypertension, hyperlipidemia, diabetes, chronic kidney disease (CKD), education duration, marriage status, employment status, annual household income, location, prostate-specific antigen (PSA), Gleason score, pathological T stage, pathological N stage and surgical margin status, they were of no difference between the RTCP + UC group and the UC group (all *P* > 0.05, Table 1).

**Table 1:** Comparison of clinical characteristics

Items	RTCP + UC (*N* = 55)	UC (*N* = 53)	*P* value
Age (years), mean ± SD	62.4 ± 9.0	63.1 ± 9.4	0.688
Smoke, No. (%)	22 (40.0)	25 (47.2)	0.452
Hypertension, No. (%)	20 (36.4)	23 (43.4)	0.455
Hyperlipidemia, No. (%)	11 (20.0)	13 (24.5)	0.571
Diabetes, No. (%)	8 (14.5)	11 (20.8)	0.397
CKD, No. (%)	9 (16.4)	10 (18.9)	0.733
Education duration (years), mean ± SD	10.0 ± 4.1	9.3 ± 4.0	0.382
Marry status, No. (%)			0.776
Single/divorced/widowed	18 (32.7)	16 (30.2)	
Married	37 (67.3)	37 (69.8)	
Employment status, No. (%)			0.785
Unemployed	36 (65.5)	36 (67.9)	
Employed	19 (34.5)	17 (32.1)	
Annual household income (¥), No. (%)			0.215
< 10,000	0 (0.0)	1 (1.9)	
10,000–29,999	20 (36.4)	13 (24.5)	
30,000–49,999	19 (34.5)	18 (34.0)	
≥ 50,000	16 (29.1)	21 (39.6)	
Location, No. (%)			0.578
Rural	15 (27.3)	12 (22.6)	
Urban	40 (72.7)	41 (77.4)	
PSA (ng/ml), No. (%)			0.194
≤ 10	19 (34.5)	14 (26.4)	
10–20	27 (49.1)	25 (47.2)	
≥ 20	9 (16.4)	14 (26.4)	
Gleason score, No. (%)			0.809
≤ 6	14 (25.5)	14 (26.4)	
7	33 (60.0)	29 (54.7)	
≥ 8	8 (14.5)	10 (18.9)	
Pathological T stage, No. (%)			0.368
pT2	34 (61.8)	28 (52.8)	
pT3	19 (34.5)	23 (43.4)	
pT4	2 (3.6)	2 (3.8)	
Pathological N stage, No. (%)			0.335
pN0	38 (69.1)	41 (77.4)	
pN1	17 (30.9)	12 (22.6)	
Surgical margin status, No, (%)			0.160
Negative	49 (89.1)	42 (79.2)	
Positive	6 (10.9)	11 (20.8)	

*RTCP* reminiscence therapy-based care program, *UC* usual care, *SD* standard deviation, *CKD* chronic kidney disease, *PSA* prostate-specific antigen

### Influence of RTCP + UC on the anxiety and depression in surgical prostate cancer patients

**Fig. 2:**
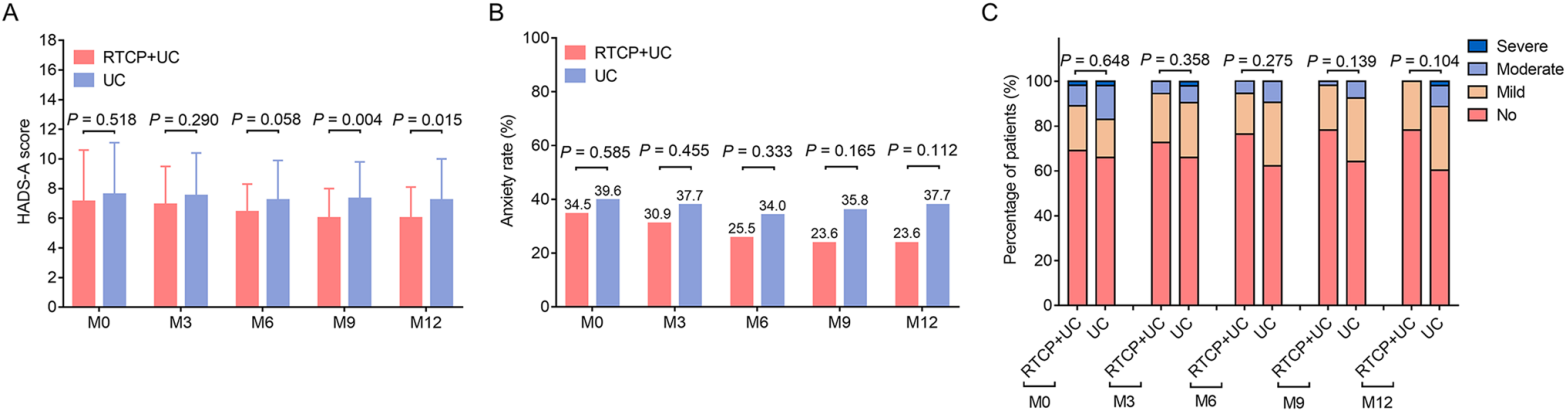
Comparison of anxiety between the RTCP + UC group and the UC group. Comparison of the HADS-A score (**A**), anxiety rate (**B**) and severity of anxiety (**C**) between the RTCP + UC group and the UC group. *HADS-A* Hospital Anxiety and Depression Scale for anxiety, *RTCP + UC* reminiscence therapy-based care program, *UC* usual care, *M* month

The HADS-A score was lower in the RTCP + UC group compared with the UC group at M9 (*P* = 0.004) and M12 (*P* = 0.015), while no difference was found between these two groups at M0, M3 or M6 (all *P* > 0.05, Fig. 2A). Concerning the influence of RTCP + UC on the anxiety rate and severity, the findings disclosed that RTCP + UC did not influence these issues at M0, M3, M6, M9 or M12 (all *P* > 0.05, Fig. 2B, C).

**Fig. 3:**
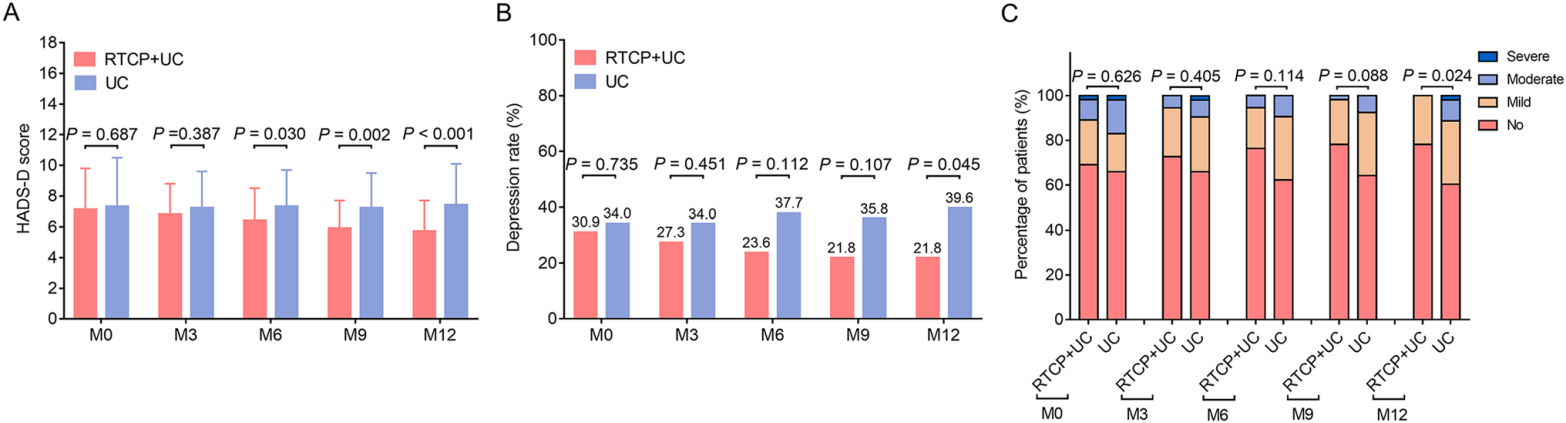
Comparison of depression between the RTCP + UC group and the UC group. Comparison of the HADS-D score (**A**), depression rate (**B**) and severity of depression (**C**) between the RTCP + UC group and the UC group. *HADS-D* Hospital Anxiety and Depression Scale for depression, *RTCP + UC* reminiscence therapy-based care program, *UC* usual care, *M* month

The HADS-D score was decreased in the RTCP + UC group than in the UC group at M6 (*P* = 0.030), M9 (*P* = 0.002) and M12 (*P* < 0.001), while no difference was observed at M0 (*P* = 0.687) or M3 (*P* = 0.387) (Fig. 3A). The depression rate in the RTCP + UC group was reduced than that in the UC group at M12 (21.8% vs. 39.6%, *P* = 0.045), while it was of no difference between these two groups at other follow-up time points (M0, M3, M6, M9) (all *P* > 0.05, Fig. 3B). Regarding the depression severity, surgical prostate cancer patients in the UC group had a more serious depression compared with those in the RTCP + UC group at M12 (*P* = 0.024). However, the depression severity showed no difference between these two groups at M0, M3, M6 and M9 (all *P* > 0.05, Fig. 3C).

### Influence of RTCP + UC on the quality of life in surgical prostate cancer patients

**Fig. 4:**
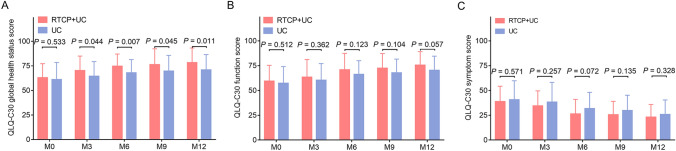
Comparison of quality of life between the RTCP + UC group and the UC group. Comparison of the QLQ-C30 global health status score (**A**), QLQ-C30 function score (**B**) and QLQ-C30 symptom score (**C**) between the RTCP + UC group and the UC group. *QLQ-C30* European Organization for Research and Treatment of Cancer quality of life Questionnaire-Core 30, *RTCP + UC* reminiscence therapy-based care program, *UC* usual care, *M* month

The QLQ-C30 global health status score was higher in the RTCP + UC group compared with the UC group at M3 (*P* = 0.044), M6 (*P* = 0.007), M9 (*P* = 0.045) and M12 (*P* = 0.011) (Fig. 4A). However, in terms of the QLQ-C30 function score and the QLQ-C30 symptom score, no difference was discovered in them between the RTCP + UC group and the UC group at any follow-up time points (M0, M3, M6, M9 and M12) (all *P* > 0.05, Fig. 4B, C).

### Influence of RTCP + UC on the survival in surgical prostate cancer patients

**Fig. 5:**
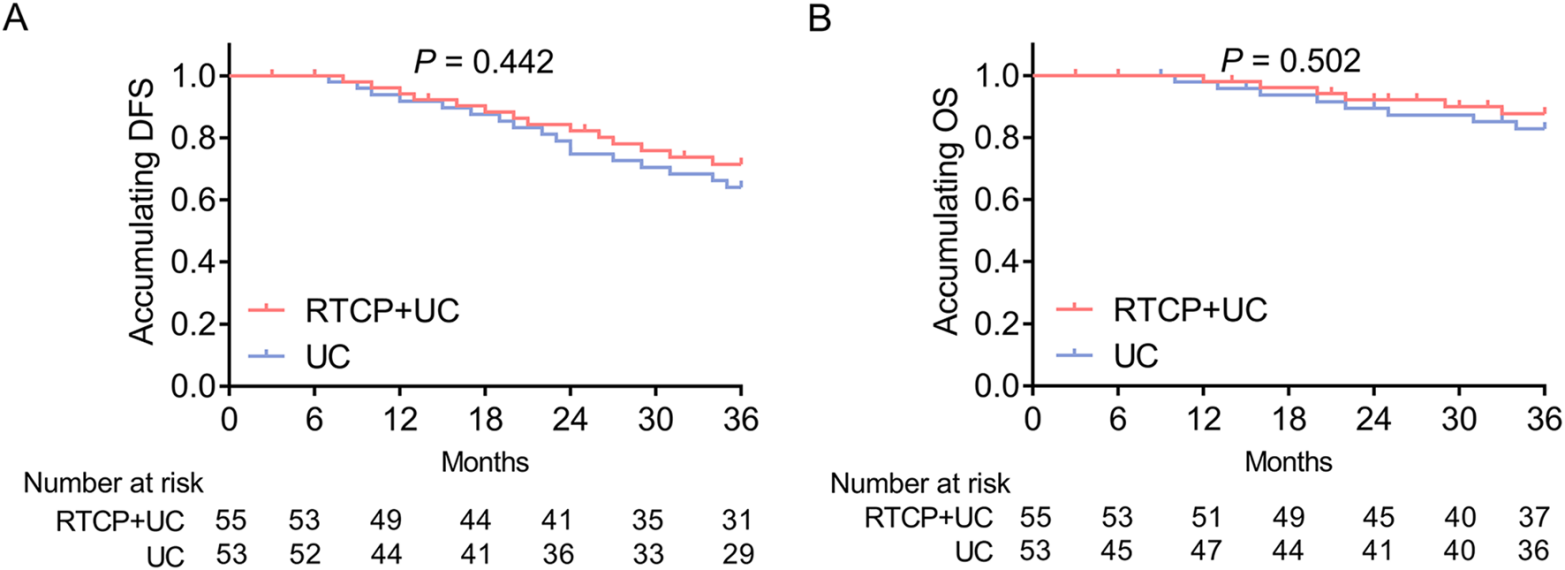
Comparison of survival between the RTCP + UC group and the UC group. Comparison of accumulating DFS (**A**) and accumulating OS (**B**) between the RTCP + UC group and the UC group. *DFS* disease-free survival, *OS* overall survival, *RTCP + UC* reminiscence therapy-based care program, *UC* usual care

As mentioned above, the occurrence of anxiety and depression might cause a worse survival in surgical prostate cancer patients; thus, we investigated the effect of RTCP + UC on the survival of surgical prostate cancer patients. Unfortunately, the finding indicated that RTCP + UC had no effect on the accumulating DFS and OS in surgical prostate cancer patients (all *P* > 0.05, Fig. 5A, B).

### Subgroup analysis

**Fig. 6:**
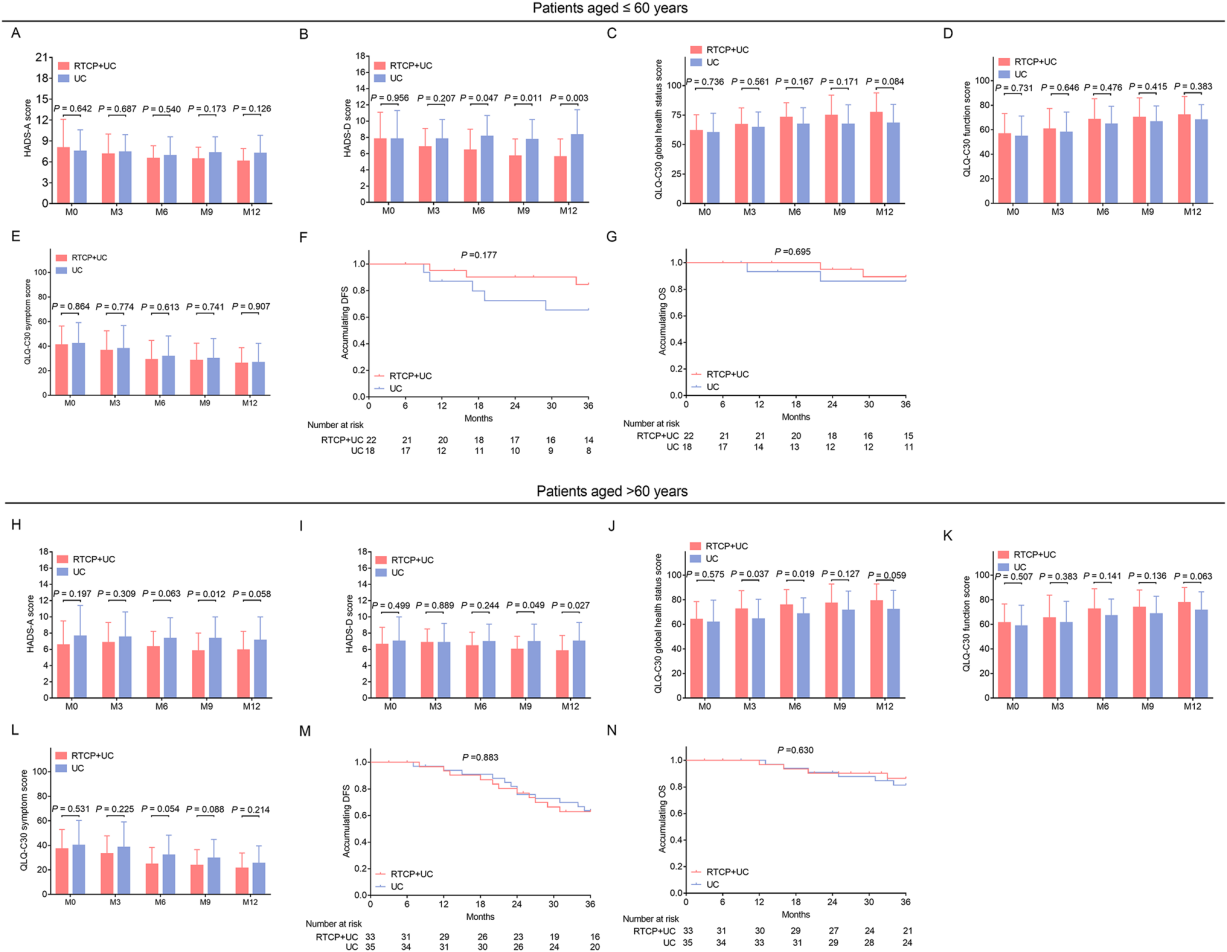
Comparison of anxiety, depression, quality of life and survival between the RTCP + UC group and the UC group in subgroups. Comparison of HADS-A score (**A**), HADS-D score (**B**), QLQ-C30 global health status score (**C**), QLQ-C30 function score (**D**), QLQ-C30 symptom score (**E**), accumulating DFS (**F**) and accumulating OS (**G**) between the RTCP + UC group and the UC group in surgical prostate cancer patients with aged ≤ 60 years; Comparison of HADS-A score (**H**), HADS-D score (**I**), QLQ-C30 global health status score (**J**), QLQ-C30 function score (**K**), QLQ-C30 symptom score (**L**), accumulating DFS (**M**) and accumulating OS (**N**) between the RTCP + UC group and the UC group in surgical prostate cancer patients with aged > 60 years. *HADS-A* Hospital Anxiety and Depression Scale for anxiety, *HADS-D* Hospital Anxiety and Depression Scale for depression, *QLQ-C30* European Organization for Research and Treatment of Cancer quality of life Questionnaire-Core 30, *DFS* disease-free survival, *OS* overall survival, *M* month, *RTCP + UC* reminiscence therapy-based care program, *UC* usual care

To determine the effect of RTCP + UC on patients with different ages, surgical prostate cancer patients were divided into two subgroups (age ≤ 60 years and age > 60 years) accordingly. The findings disclosed that the effect of RTCP + UC was similar between patients with age ≤ 60 years and patients with age > 60 years (Fig. 6A–N).

To further determine that whether the effect of RTCP + UC on patients was influenced by the comorbidities and tumor features, the subgroups analysis was performed. It seems that the influence of RTCT on anxiety, depression and quality of life is more obvious in patients without hypertension, hyperlipidemia, and diabetes, meanwhile in patients with Gleason score ≥ 7 and negative surgical margin (all *P* < 0.05, Supplementary Tables 1 and 2).

## Discussion

Reminiscence therapy is a nursing intervention modality that is firstly generated for the treatment of cognition impairment in patients with Alzheimer’s disease and dementia [20, 21]. For instance, a multicenter randomized controlled study discloses that in older adults with Alzheimer’s disease and vascular dementia, the reminiscence therapy may decrease the global cognitive function (MMSE) score but increase the memory (MAT) score compared with usual care [20]. With the wide application of reminiscence therapy in other brain-related diseases, such as acute ischemic stroke, reminiscence therapy is also reported to serve as an efficient strategy in attenuating mood disorders, such as anxiety and depression, apart from ameliorating the cognitive function [12, 22]. As for the application of reminiscence therapy on nursing care in cancer patients, relevant studies are still lacking. What’s more, the limited published studies indicate that reminiscence therapy might alleviate anxiety and depression, improve the quality of life in colorectal cancer patients and non-small cell lung cancer patients [13, 14]. However, no study performed on the effect of reminiscence therapy on anxiety, depression, quality of life and survival in surgical prostate cancer patients. Our study found that RTCP + UC eased anxiety and depression in surgical prostate cancer patients. Possible explanations are as follows: (1) RTCP + UC was conducted through recalling the memory of surgical prostate cancer patients, which might help surgical prostate cancer patients build the confidence of fighting against disease, overcome the difficulties caused by the complications of the disease and subsequent therapy; therefore, RTCP + UC alleviated the anxiety of surgical prostate cancer patients; (2) except recalling memory, RTCP + UC also encouraged patients to share funny childhood things or past experiences to others, which might help surgical prostate cancer patients keep away from gloomy mood during the communication process; thereby, RTCP + UC ameliorated the depression status; (3) surgical prostate cancer patients might feel “be cared” or “be loved” during the RTCP + UC period. Therefore, RTCP + UC might bring a happy mood to the surgical prostate cancer patients to remit the anxiety and depression situation; (4) RTCP + UC might decrease the level of neurotransmitters which are involved in the pathogenesis of anxiety and depression, such as 5-hydroxytryptamine, dopamine or norepinephrine. However, this hypothesis needed further exploration.

Meanwhile, it has been shown that reminiscence therapy can improve the quality of life in colorectal cancer patients and non-small cell lung cancer patients [13, 14]. For instance, reminiscence therapy may enhance the QLQ-C30 global health status score in post-operational non-small cell lung cancer patients [13]. Our study found that RTCP + UC might upregulate the QLQ-C30 global health status score in surgical prostate cancer patients, and this finding was similar with previous studies [13, 14]. This phenomenon might be explained by that RTCP + UC might reduce anxiety and depression of surgical prostate cancer patients, which further led to a more energetic attitude toward life with a strong eager to live, sport and work in surgical prostate cancer patients. Furthermore, these patients achieved a better global health status score.

As illustrated by previous studies, the occurrence of anxiety and depression may lead to poor survival in surgical prostate cancer patients, and we hypothesized that the RTCP + UC might elevate the survival profile of surgical prostate cancer patients through alleviating the anxiety and depression status [9]. Unfortunately, in line with previous studies, we did not observe any difference in DFS or OS between the RTCP + UC group and the UC group [13, 14]. Explanations could be that the total follow-up period was 3 years, while the influence of RTCP + UC on the survival profile of surgical prostate cancer patients needed a longer follow-up period.

Apart from the main findings in this study, the discoveries from subgroups analysis are also of great interest. In details, we found that the influence of RTCT + UC on anxiety, depression and quality of life was more obvious in patients with Gleason score ≥ 7 and negative surgical margin, which could be explained as follow: The tendency for reduction of HADS-A, HADS-D score and elevation of QLQ-C30 score in RTCT + UC group could also be observed in patients with Gleason score < 7 and positive surgical margin, while the sample sizes in patients with Gleason score < 7 [only 14 (25.5%) and 14 (26.4%) patients with Gleason score < 7 in RTCT + UC group and UC group, respectively] and positive surgical margin [only 6 (10.9%) and 11 (20.8%) patients with positive surgical margin in RTCT + UC group and UC group, respectively] were small; thus, this might be the reason for why the significant statistical differences of HADS-A, HADS-D score and QLQ-C30 score between RTCT + UC group and UC group were only observed in patients with Gleason score ≥ 7 and negative surgical margin, but not in those with Gleason score < 7 or positive surgical margin.

There were some limitations in the present study: (1) the sample size was still relatively small, although it was sufficient based on the calculation of the minimal sample size, which might cause low statistical power; (2) The follow-up period was relatively short, which might cause unsatisfactory results in the survival profiles and some other aspects; (3) The detailed influence of RTCP + UC on anxiety and depression was still unclear, which needed further exploration; (4) In RTCP + UC group, the nurses had more chance to contact with the patients compared with the nurses in UC group; thus, this might be a potential cofounding factor which affected the findings in this study.

To be conclusive, RTCP + UC serves as an optional nursing modality in alleviating anxiety and depression, improving quality of life in surgical prostate cancer patients.

## Supplementary Information

Below is the link to the electronic supplementary material.

Supplementary file1 (DOCX 21 KB)

Supplementary file2 (DOCX 21 KB)
