# Supplementary material for: Reminiscence therapy-based care program serves as an optional nursing modality in alleviating anxiety and depression, improving quality of life in surgical prostate cancer patients
Source: Int Urol Nephrol. 2022 Jul 16;54(10):2467–76. doi: 10.1007/s11255-022-03282-6 (PMC9463279; doi:10.1007/s11255-022-03282-6)
Supplement: Supplementary file 1 — Supplementary file1 (DOCX 21 KB) [file 11255_2022_3282_MOESM1_ESM.docx]

**Supplementary table 1.** Subgroup analysis based on comorbidities

| Items | **HADS-A score (M12)** | **HADS-D score (M12)** | **QLQ-C30 score (M12),** **Mean±SD** | | |
| --- | --- | --- | --- | --- | --- |
|  | Mean±SD | Mean±SD | Global health status | Function | Symptom |
| **Patients with hypertension** |  |  |  |  |  |
| RTCP+UC | 6.2 ± 2.4 | 6.6 ± 2.0 | 74.1 ± 12.6 | 71.9 ± 12.5 | 24.6 ± 11.6 |
| UC | 7.1 ± 2.9 | 7.4 ± 2.9 | 72.8 ± 13.3 | 72.0 ± 13.3 | 22.7 ± 12.5 |
| *P* value | 0.259 | 0.303 | 0.750 | 0.980 | 0.618 |
| **Patients** **without hypertension** |  |  |  |  |  |
| RTCP+UC | 5.9 ± 1.9 | 5.3 ± 1.9 | 80.7 ± 15.7 | 77.3 ± 14.2 | 22.2 ± 13.5 |
| UC | 7.2 ± 2.8 | 7.5 ± 2.6 | 69.2 ± 17.3 | 68.7 ± 15.2 | 27.9 ± 16.1 |
| *P* value | 0.028 | <0.001 | 0.006 | 0.022 | 0.123 |
| **Patients with hyperlipidemia** |  |  |  |  |  |
| RTCP+UC | 5.4 ± 2.6 | 6.6 ± 2.2 | 82.6 ± 7.9 | 79.3 ± 10.8 | 23.7 ± 10.2 |
| UC | 7.2 ± 2.7 | 8.3 ± 3.7 | 73.9 ± 18.2 | 72.5 ± 19.6 | 22.0 ± 14.7 |
| *P* value | 0.113 | 0.179 | 0.157 | 0.316 | 0.747 |
| **Patients without** **hyperlipidemia** |  |  |  |  |  |
| RTCP+UC | 6.1 ± 2.0 | 5.6 ± 1.9 | 77.3 ± 16.1 | 74.4 ± 14.3 | 22.9 ± 13.5 |
| UC | 7.2 ± 2.9 | 7.2 ± 2.2 | 69.8 ± 14.9 | 69.4 ± 12.4 | 26.9 ± 14.7 |
| *P* value | 0.060 | 0.001 | 0.029 | 0.094 | 0.201 |
| **Patients with diabetes** |  |  |  |  |  |
| RTCP+UC | 6.8 ± 2.9 | 6.3 ± 2.3 | 78.0 ± 12.5 | 79.9 ± 11.3 | 28.1 ± 12.7 |
| UC | 6.9 ± 3.2 | 6.7 ± 2.3 | 75.1 ± 17.0 | 75.1 ± 16.6 | 18.7 ± 12.3 |
| *P* value | 0.914 | 0.657 | 0.688 | 0.490 | 0.124 |
| **Patients without** **diabetes** |  |  |  |  |  |
| RTCP+UC | 5.9 ± 1.9 | 5.7 ± 2.0 | 78.4 ± 15.4 | 74.6 ± 14.1 | 22.2 ± 12.8 |
| UC | 7.2 ± 2.7 | 7.6 ± 2.7 | 69.6 ± 15.3 | 68.9 ± 13.7 | 27.5 ± 14.9 |
| *P* value | 0.007 | <0.001 | 0.009 | 0.055 | 0.075 |
| **Patients with CKD** |  |  |  |  |  |
| RTCP+UC | 6.4 ± 1.4 | 5.3 ± 1.4 | 86.2 ± 7.4 | 86.2 ± 8.8 | 19.6 ± 13.0 |
| UC | 7.1 ± 1.6 | 8.3 ± 3.5 | 68.4 ± 14.0 | 70.9 ± 11.8 | 25.9 ± 14.3 |
| *P* value | 0.360 | 0.029 | 0.003 | 0.005 | 0.328 |
| **Patients without CKD** |  |  |  |  |  |
| RTCP+UC | 5.9 ± 2.2 | 5.8 ± 2.1 | 76.8 ± 15.5 | 73.2 ± 13.6 | 23.7 ± 12.8 |
| UC | 7.2 ± 3.0 | 7.2 ± 2.4 | 71.3 ± 16.1 | 70.0 ± 15.0 | 25.6 ± 15.0 |
| *P* value | 0.025 | 0.005 | 0.106 | 0.285 | 0.528 |

RTCP, reminiscence therapy-based care program; UC, usual care; SD, standard deviation; CKD, chronic kidney disease; HADS-A, Hospital Anxiety and Depression Scale for anxiety; HADS-D, Hospital Anxiety and Depression Scale for depression; QLQ-C30, European Organization for Research and Treatment of Cancer quality of life Questionnaire-Core 30.
