# Supplementary material for: Reminiscence therapy-based care program serves as an optional nursing modality in alleviating anxiety and depression, improving quality of life in surgical prostate cancer patients
Source: Int Urol Nephrol. 2022 Jul 16;54(10):2467–76. doi: 10.1007/s11255-022-03282-6 (PMC9463279; doi:10.1007/s11255-022-03282-6)
Supplement: Supplementary file 2 — Supplementary file2 (DOCX 21 KB) [file 11255_2022_3282_MOESM2_ESM.docx]

**Supplementary table 2.** Subgroup analysis based on tumor features

| Items | **HADS-A score (M12)** | **HADS-D score (M12)** | **QLQ-C30 score (M12),** **Mean±SD** | | |
| --- | --- | --- | --- | --- | --- |
|  | Mean±SD | Mean±SD | Global health status | Function | Symptom |
| **Patients with Gleason score <7** |  |  |  |  |  |
| RTCP+UC | 6.4 ± 2.7 | 6.6 ± 1.9 | 75.6 ± 15.5 | 74.4 ± 12.8 | 28.5 ± 9.7 |
| UC | 7.1 ± 3.6 | 7.4 ± 2.3 | 65.4 ± 17.2 | 67.4 ± 15.5 | 27.6 ± 15.4 |
| *P* value | 0.555 | 0.291 | 0.113 | 0.203 | 0.850 |
| **Patients with Gleason score ≥7** |  |  |  |  |  |
| RTCP+UC | 5.9 ± 1.9 | 5.5 ± 2.0 | 79.3 ± 14.7 | 75.7 ± 14.2 | 21.2 ± 13.3 |
| UC | 7.2 ± 2.5 | 7.4 ± 2.8 | 72.7 ± 14.9 | 71.1 ± 14.0 | 25.0 ± 14.6 |
| *P* value | 0.009 | 0.001 | 0.050 | 0.154 | 0.230 |
| **Patients with pT2 stage** |  |  |  |  |  |
| RTCP+UC | 5.7 ± 2.0 | 5.9 ± 2.0 | 79.9 ± 12.6 | 76.2 ± 12.6 | 22.5 ± 11.6 |
| UC | 6.8 ± 2.7 | 7.5 ± 2.9 | 73.8 ± 14.7 | 73.0 ± 13.8 | 25.3 ± 15.2 |
| *P* value | 0.057 | 0.013 | 0.083 | 0.337 | 0.406 |
| **Patients with pT3-4 stage** |  |  |  |  |  |
| RTCP+UC | 6.5 ± 2.3 | 5.5 ± 2.1 | 75.8 ± 18.1 | 74.0 ± 15.7 | 24.0 ± 14.8 |
| UC | 7.5 ± 2.9 | 7.4 ± 2.4 | 67.4 ± 16.4 | 67.0 ± 14.7 | 26.0 ± 14.5 |
| *P* value | 0.190 | 0.008 | 0.104 | 0.126 | 0.641 |
| **Patients with pN0 stage** |  |  |  |  |  |
| RTCP+UC | 6.2 ± 2.3 | 5.9 ± 2.0 | 77.4 ± 15.0 | 74.2 ± 14.8 | 23.9 ± 12.4 |
| UC | 7.0 ± 2.7 | 7.4 ± 2.8 | 71.2 ± 15.5 | 70.2 ± 14.2 | 26.4 ± 14.9 |
| *P* value | 0.168 | 0.005 | 0.073 | 0.217 | 0.423 |
| **Patients with pN1 stage** |  |  |  |  |  |
| RTCP+UC | 5.5 ± 1.4 | 5.5 ± 2.1 | 80.4 ± 14.7 | 77.9 ± 11.0 | 21.2 ± 13.9 |
| UC | 7.8 ± 3.2 | 7.4 ± 2.4 | 69.4 ± 17.1 | 70.2 ± 15.6 | 23.2 ± 14.5 |
| *P* value | 0.016 | 0.029 | 0.075 | 0.125 | 0.711 |
| **Patients with negative surgical margin** |  |  |  |  |  |
| RTCP+UC | 5.9 ± 2.0 | 5.7 ± 2.1 | 77.9 ± 15.5 | 74.5 ± 14.0 | 22.5 ± 13.1 |
| UC | 6.9 ± 2.9 | 7.3 ± 2.3 | 70.2 ± 15.6 | 70.1 ± 14.2 | 26.5 ± 15.0 |
| *P* value | 0.055 | 0.001 | 0.022 | 0.139 | 0.177 |
| **Patients with positive** **surgical margin** |  |  |  |  |  |
| RTCP+UC | 6.3 ± 3.3 | 6.3 ± 1.5 | 82.0 ± 8.3 | 82.5 ± 9.7 | 27.7 ± 9.8 |
| UC | 8.0 ± 2.4 | 8.1 ± 3.7 | 72.7 ± 16.7 | 70.5 ± 15.8 | 22.5 ± 13.8 |
| *P* value | 0.243 | 0.288 | 0.225 | 0.112 | 0.428 |

RTCP, reminiscence therapy-based care program; UC, usual care; SD, standard deviation; HADS-A, Hospital Anxiety and Depression Scale for anxiety; HADS-D, Hospital Anxiety and Depression Scale for depression; QLQ-C30, European Organization for Research and Treatment of Cancer quality of life Questionnaire-Core 30.
